# Prenatal Alcohol Exposure Increases Postnatal Acceptability of Nicotine Odor and Taste in Adolescent Rats

**DOI:** 10.1371/journal.pone.0102255

**Published:** 2014-07-16

**Authors:** Nicole M. Mantella, Steven L. Youngentob

**Affiliations:** 1 Department of Psychiatry and Behavioral Sciences, State University of New York Upstate Medical University, Syracuse, New York, United States of America; 2 State University of New York Developmental Exposure Alcohol Research Center, Syracuse & Binghamton, New York, United States of America; Barnard College, Columbia University, United States of America

## Abstract

Human studies indicate that alcohol exposure during gestation not only increases the chance for later alcohol abuse, but also nicotine dependence. The flavor attributes of both alcohol and nicotine can be important determinants of their initial acceptance and they both share the component chemosensory qualities of an aversive odor, bitter taste and oral irritation. There is a growing body of evidence demonstrating epigenetic chemosensory mechanisms through which fetal alcohol exposure increases adolescent alcohol acceptance, in part, by decreasing the aversion to alcohol's bitter and oral irritation qualities, as well as its odor. Given that alcohol and nicotine have noteworthy chemosensory qualities in common, we investigated whether fetal exposure to alcohol increased the acceptability of nicotine's odor and taste in adolescent rats. Study rats were alcohol-exposed during fetal development via the dams' liquid diet. Control animals received *ad lib* access to an iso-caloric, iso-nutritive diet throughout gestation. Odorant-induced innate behavioral responses to nicotine odor (*Experiment 1*) or orosensory-mediated responses to nicotine solutions (*Experiment 2*) were obtained, using whole-body plethysmography and brief access lick tests, respectively. Compared to controls, rats exposed to fetal alcohol showed an enhanced nicotine odor response that was paralleled by increased oral acceptability of nicotine. Given the common aversive component qualities imbued in the flavor profiles of both drugs, our findings demonstrate that like postnatal alcohol avidity, fetal alcohol exposure also influences nicotine acceptance, at a minimum, by decreasing the aversion of both its smell and taste. Moreover, they highlight potential chemosensory-based mechanism(s) by which fetal alcohol exposure increases the later initial risk for nicotine use, thereby contributing to the co-morbid expression with enhanced alcohol avidity. Where common chemosensory mechanisms are at play, our results suggest broader implications related to the consequence of fetal exposure with one substance of abuse and initial acceptability of others.

## Introduction

It is well accepted that human studies reveal: (a) a strong association between exposure to alcohol during gestation and the increased probability for alcohol abuse during the vulnerable period of adolescence [1]–[4]; and (b) that the younger the first experience, the higher the chance of continuing abuse [2], [5]. There is also evidence that prenatal alcohol exposure is a risk factor for nicotine dependence, as well [6], [7].

Alcohol consumption and the use of tobacco products are known to be highly correlated behaviors [8]–[11] and co-morbid dependence is common [12]–[14]. Indeed, the incidence of smoking among alcoholics as compared to non-alcoholics is quite high [8], [15], [16]. For example, in a case-control study of alcoholics versus non-alcoholics, DiFranza and Guerrera [15] showed a difference of 83 versus 34%, respectively. They also found that compared to children in general, those who became alcoholics later in life had a higher probability of becoming tobacco product abusers. Jackson and colleagues [17] found that initiation and persistence of smoking in adolescence varied as a function of alcohol use. Increases in alcohol and tobacco use showed a monotonic relationship during adolescence and through young adulthood.

Several factors are thought to contribute to the co-morbid relationship between alcohol and tobacco dependence. They include, but are not limited to socio-cultural factors [18], shared genetic influences [19] and, at a neurobiological level, pharmacologic cross-tolerance [20]. Despite studies investigating the possible basis for alcohol and nicotine interactions in both rodents and humans (see rev [21]), the unifying underlying mechanism(s) for the effect of fetal alcohol exposure, per se, on both postnatal alcohol and nicotine acceptance is essentially not known. In terms of other neurobiological synergies that have been suggested as a basis for alcohol and nicotine co-morbidity [21], [22], there is evidence that prenatal alcohol exposure impacts many of the same molecular and cellular targets influenced by nicotine: namely, the central catecholamine receptor systems [23]–[26]. Nicotine and alcohol both stimulate the mesocorticolimbic dopamine system and this, in turn, promotes intake and reinforcement [21]. Like nicotine, there is also evidence that alcohol interacts with nicotinic cholinergic receptors [22], [27]–[29]. In short, there is the potential for a complex interaction via which enhanced alcohol avidity, as a consequence of prenatal alcohol exposure [1], [3], [4] *once initiated*, could contribute to the observed link with smoking behavior [6], [7].

The above notwithstanding, one basic question related to prenatal alcohol exposure arises: are there potential mechanisms by which prior fetal alcohol exposure could directly impact initial choice and intake behavior for both drugs? The flavor attributes (viz., the sensory integration of smell, taste and oral irritation) of both alcohol [30] and nicotine [31], [32] are key contributors to their acceptance. With specific regard to alcohol, animal studies have demonstrated there are epigenetic chemosensory mechanisms via which a mother's use of alcohol during pregnancy is potentially transmitted to her children. Using a rat model, fetal exposure has been shown to increase alcohol acceptability in adolescent animals by decreasing the normally aversive flavor attributes of alcohol's quinine-like bitter taste, capsaicin-like oral burning sensation and aversive odor properties [33], [34]. In accordance with the orosensory mediated behavioral findings, fetal alcohol exposure has also been shown to decrease the expression of bitter (*T2rs:* in particular, those sensing quinine) and oral irritation (in particular, *Trpv1*) receptor genes, basic to alcohol flavor perception in adolescent rats [35]. In addition, an observed decreased expression of *Trpm5*, a receptor important to calcium channel opening during the transduction of bitter, sweet and umami, likely contributes to this process, as well. Fetal exposure in rats also specifically decreased the expression of *T2r38*, a bitter receptor that responds to phenylthiocarbamide [PTC] and 6-n-propylthiouracil [PROP] and one that has been implicated in human alcohol (see rev [30]) and nicotine [36]–[39] acceptance.

With respect to odor, fetal alcohol exposure has been shown to alter the expression of genes important for synaptic transmission, plasticity and neuronal development in the olfactory bulbs of adolescent animals [40]. Alternative splicing analysis also revealed genes related to addictive behavior, synaptic plasticity and DNA repair [41]. Finally, the observation of decreased *T2r*, *Trpv1* and *Trpm5* gene expression in the oral cavity may likely generalize to the nasal cavity where both *Trpv1* (nasal trigeminal: e.g., [42], [43]) and, solitary chemosensory cells expressing *T2r and Trpm5* [44]–[46] respond to inhaled irritants.

Nicotine has several component chemosensory qualities in common with alcohol. Notably, nicotine, itself, plays a prominent role in determining the smell and flavor quality of tobacco smoke [32] and this, by extension, influences smoker enjoyment [31], [47]. With respect to smell, along with the positive attributes of warm and sweet, nicotine is also described as irritating and aversive [48]–[51]. Both nasal chemesthesis and odor conveyed through the nasal trigeminal and olfactory systems, respectively, are fundamental to the perceptual process (e.g., [42], [43], [48], [50]–[52]). *T2r- and Trpm5*-expressing solitary chemosensory cells in the nasal cavity also respond to nicotine [44], thereby suggesting a role in this process.

With respect to orosensory-mediated perception, nicotine has been described as both irritating [53], [54] and having a bitter taste [37]. Not surprisingly, like alcohol, oral irritation in response to nicotine is conveyed through the trigeminal system (e.g., [53], [54]). Nicotine concentrations consistent with those found in the saliva of people using tobacco products or oral treatment aids to ameliorate smoking (e.g., electronic cigarettes) sensitize/activate both *Trpv1* and *Trpa1* channels [55], although only the former sensory channel has been implicated in alcohol exposure and acceptance [34], [35], [56]. The gustatory response to nicotine taste is potentially mediated through several pathways [57]. In common with the response to fetal alcohol are those mediated both through a *Trpm5* dependent pathway (*ibid*) and specific bitter receptors (i.e., *T2r*). With regard to the latter, several lines of evidence demonstrate that bitter taste is highly relevant to smoking behavior. For example, the ability to appreciate the bitter taste of PTC and PROP, a trait determined by genetic variations of the bitter taste receptor gene *T2R38* [58], protects against cigarette smoking [36]–[39]).

Given this prior body of work, we tested whether fetal alcohol exposure altered the (a) odorant-induced innate behavioral response to nicotine odor and (b) orosensory-mediated acceptability of nicotine.

## Materials and Methods

### Ethics Statement

All procedures were approved by SUNY Upstate Medical University's IACUC (Institutional Animal Care and Use Committee) (PHS Assurance: A3514-01). Where applicable, appropriate procedures were used to minimize pain or distress.

### General Overview

In this study we evaluated the: (a) odorant-induced behavioral responses to nicotine odor or (b) orosensory-mediated responses to nicotine and sucrose solutions of prenatal alcohol or control exposed rats, using whole-body plethysmography and brief access lick tests. Animals were tested between postnatal (P) day 28 and 35 (*see details below*). These ages were chosen because previous studies examining the consequences of fetal experience with alcohol on the response to the drug's flavor attributes demonstrated a decreased aversion to the component qualities of odor, bitter and oral irritation that persisted into adolescence (e.g., [33], [34], [40], [59], [60]). Importantly, given that nicotine shares the same aversive qualities as alcohol [37], [53], [54] and smoking is co-morbidly expressed with alcohol consumption in humans [8], [10], these ages permitted us to test the hypothesis that prenatal alcohol experience would alter the postnatal behavioral response to nicotine in adolescence (P28–P42: e.g., [61]).

### Prenatal Alcohol Exposure

On fetal development (G) day 5, pregnant Long-Evans Hooded females (Harlan Labs, Indianapolis, IN) were weighed, separated into groups containing two weight-matched dams and then randomly assigned to one of two maternal exposure groups. Alcohol exposed dams (ET) received an *ad-lib* liquid diet (L10251, Research Diets, NJ) that provided 35% of their daily calories via alcohol during G11–20, subsequent to gradual exposure to lower concentrations of the diet beginning on G6. This approach to alcohol exposure yields peak blood concentration levels of approximately 150 mg/dl when samples are taken 3 hr after lights out in the vivarium on G17 [62]–[65]. This standard method for exposing dams (e.g., [64]–[66]) provided alcohol during a time period of olfactory, gustatory and oro-somatosensory system development (G11–20) when: (1) dietary manipulations modify taste receptor cell transduction (e.g., [67]–[70]) and (2) fetal alcohol exposures alter the odor and orosensory responses to alcohol and surrogates of its component flavor attributes of bitter and oral irritation [33], [34].

One dam within a block served as control, namely, a free-choice liquid animal (FCL). FCL dams were provided *ad lib* access to a liquid diet that was iso-caloric and iso-nutritive (L10252; Research Diets, NJ) to that fed to the ET dams. In the control diet maltose dextrin was substituted for the calories provided by alcohol. It should be noted that we only used one control group, namely, *ad lib* access dams, as previously we have shown no difference in the odor-mediated, orosensory or alcohol avidity responses of offspring resulting from pair-fed (i.e., a control for reduced diet intake in ET dams) versus *ad lib* access animals (e.g., [33], [65], [71]). As highlighted in Table 1, on balance the offspring of alcohol and *ad lib* exposed dams do not demonstrably differ across eight characteristics. The eight characteristics,were: (1) pups/litter, (2) males/litter, (3) females/litter, (4) litter weight, (5) male weight on day 1 of brief access lick training, (6) female weight on day 1 of brief access lick training, (7) male weight on the day of odor testing and (8) female weight on the day of odor testing. Only female weight on day 1 of brief access lick training differed as a function of maternal treatment (t [20] = 3.34, Bonferonni corrected *p*<0.05),

**Table 1:** Characteristics of Offspring.

Maternal Exposure	Pups/Litter	Males/Litter	Female/Litter	Litter Weight	Male Weight Day 1 Brief Access Training	Female Weight Day 1 Brief Access Training	Male Weight at Odor Testing	Female Weight at Odor Testing
Alcohol	12.0 +/−0.72	6.09+/−0.57	5.09+/−0.47	68.8+/−3.56	59.4+/−1.24	56.0+/−* 1.00	74.7+/−3.04	67.4+/−3.47
Control	12.2+/−0.29	6.72+/−0.52	5.45+/−0.36	77.7+/−2.29	62.7+/−1.77	60.9+/−1.07	75.2+/−2.3	68.9+/−1.81

Weights = grams. The data represent the mean +/− se.

* = *P*<0.05.

All dams were provided Nylabones (Nylabone Products, Neptune City, NJ) to facilitate the need to chew in the absence of a chow diet.

Within 24 hours of being born litters were reduced to 10 and transferred to *ad lib* food and water fed dams.

### Experimental Subjects

To determine whether fetal alcohol exposure altered the odor-mediated behavioral responses to nicotine odor, one animal of both genders was randomly selected from any particular litter within a dyad of ET and FCL dams. The progeny from a total of 10 dyads were used for the olfactory behavioral testing, resulting in 20 ET (10 male, 10 female) and 20 FCL (10 male, 10 female) animals. For the orosensory-mediated evaluations the progeny of 11 dyads of ET and FCL dams were used. Progeny were randomly selected as above, yielding 22 ET (11 male, 11 female) and 22 FCL (11 male, 11 female) animals.

### Experiment 1: Innate Behavioral Response to Nicotine Odor

We applied an unbiased method for testing the inborn responsiveness to specific odorant stimuli in rodents, the theory and explicit details of which have been described (e.g., [33], [65], [72], [73]). In summary of this approach, whole-body plethysmography was used to monitor the inherent sniffing responses (i.e., respiratory airflow patterns) following the delivery of air or different concentrations of the odorant nicotine. Odorant stimuli were delivered into a testing chamber with a continuous airflow using a flow-dilution olfactometer (*ibid*). A computer monitored and controlled the behavioral testing, the generation of stimuli, and the gathering of sniffing data.

P35 male and female ET and FCL animals were tested in randomized order. A testing session for any given animal proceeded as follows: (1) an habituation period of forty air only trials and (2) following the habituation trials, the presentation of five different concentrations of nicotine odor in an ascending series. The nicotine concentration series was 3.125×10^−3^, 6.25×10^−3^, 1.25×10^−2^, 2.5×10^−2^ and 5×10^−2^ (concentration is expressed as the fraction of vapor saturation at 20°C) [73]. For each concentration of odorant presented, an animal received 20 trials of the randomized presentation of 10 air and 10 odorant stimuli.

The examination of the recorded sniffing responses proceeded according to our previous analytic approach (e.g., [33], [59], [60], [65], [73], [74]). Briefly, the airflow patterns generated by the animals' innate sniffing responses to odorant were initially broken down by computer evaluation of the patterns into 14 different respiratory measures (i.e., response dimensions) (e.g., [59], [60], [65], [73], [74]). Following our prior approach (*ibid*), the 14 measures were: sniff frequency; the number of inspiratory and expiratory sniffs; the duration, volume, average flow rate, and peak flow rate of an inspiratory and expiratory sniff; the total inspiratory and expiratory volume; and the total apneic duration. Therefore, each ET and FCL animal provided 14 response variables for every one of the five concentrations tested. These variables were used to create an “Index” that quantified the animals' odor-mediated behavioral responses that, in turn, were used to test experimental main effects (*ibid*).

In the first step of creating an “Index” we used a standard principle components analysis (PCA). This compressed the 14 variables for each response to odor of an animal into two orthogonal values (namely, factor 1 and 2 of the PCA) (N.B.: *a priori* we focused our assessment to those PCA factors with Eigen values greater than a Kaiser criterion of 1 [73], [74]). As such, the animals' 14×5 data matrices were compressed to a set of 2-PCA factors×5-concentration matrices. Next, to create a behavioral index for every rat that integrated the animals' responses for every concentration of nicotine evaluated, separate analyses were performed on each PCA factor, using multivariate linear regression. Here the odor-mediated behavioral response values for the five concentrations of nicotine served as the dependent variables and gestational exposure as the independent variable. Each of the regression analyses, in turn, provided the coefficients for each concentration of nicotine for the respective PCA factors. The derived index value from each PCA factor for a given animal was the total of the regression constant from the analysis plus the individual PCA value at each concentration of nicotine multiplied by the applicable coefficient. This resulted in x and y pairs of data that were used to place each rat in a nicotine odorant stimulus, behavioral response space (*see* *Results*).

### Experiment 2: Assessment of Taste Responsiveness to Nicotine

We evaluated the taste responsiveness of ET and FCL animals to nicotine and the appetitive tastant sucrose. Because of the aversive nature of nicotine we first tested all animals on sucrose to provide experience with the task. In this latter respect, previous studies have shown no effect of fetal alcohol exposure on taste responsiveness to this stimulus [33]. To accomplish this, we used standard brief-access taste tests and an automated gustometer (Dilog Instruments, Inc., Tallahassee, FL) that presented tastants following a programmed schedule, and monitored licking responses during 10 s trials (e.g., [33], [34], [75]). Each animal received 3 days of training beginning on P25.

Briefly, prior to training, the rats were deprived of water for 22.5 hr. All training sessions were 30 min. On the first training day, animals were permitted to drink de-ionized (DI) water without restriction from a stationary sipper tube for 30 min, and then returned to their cages where they received 1 hr of *ad lib* access to water and food. The animals were then deprived of water for another 22.5 hr (with continuous access to food). On the second day of training, the animals had access to the DI sipper tubes during 10 s trials. Each trial was separated by a 7.5 s inter-trial interval. A computer-activated shutter controlled access to the sipper tubes. Following this session, the rats were again deprived of water for another 22.5 hr. On the third training day, the same procedure as in day two was used.

Following training, the rats participated in a single 30-min test session for a concentration series of sucrose (0.03, 0.1, 0.2, 0.3, 0.6 M) [33] and nicotine (0.1, 0.3, 1.0, 3.0, 6.0 mM) [57] on different days, using the last testing parameters outlined above. DI water was also included as a stimulus in each session. For a given tastant, the order of presentation for the different concentrations was pseudo-randomized using a balanced Latin Square design. Further, for each block of six trials every concentration of a taste stimulus and DI water was presented once without replacement before the start of the next block.

As noted, for all animals the order of tastant testing was sucrose (on P28) followed by nicotine (on P31). The two testing days were separated by a recovery day where food and water were provided *ad lib*. To encourage licking for the sucrose solutions, rats were deprived of food for 22.5 hr prior to testing. By contrast, to encourage licking for the nicotine, the rats were deprived of water for 22.5 hr prior to testing.

For each tastant we determined the average number of licks and latency to first lick to each stimulus concentration across the entire test session.

## Results

### Consequence of Fetal Alcohol Exposure on the Chemosensory Response to Nicotine

#### Experiment 1: Response to Nicotine Odor

**Figure 1:**
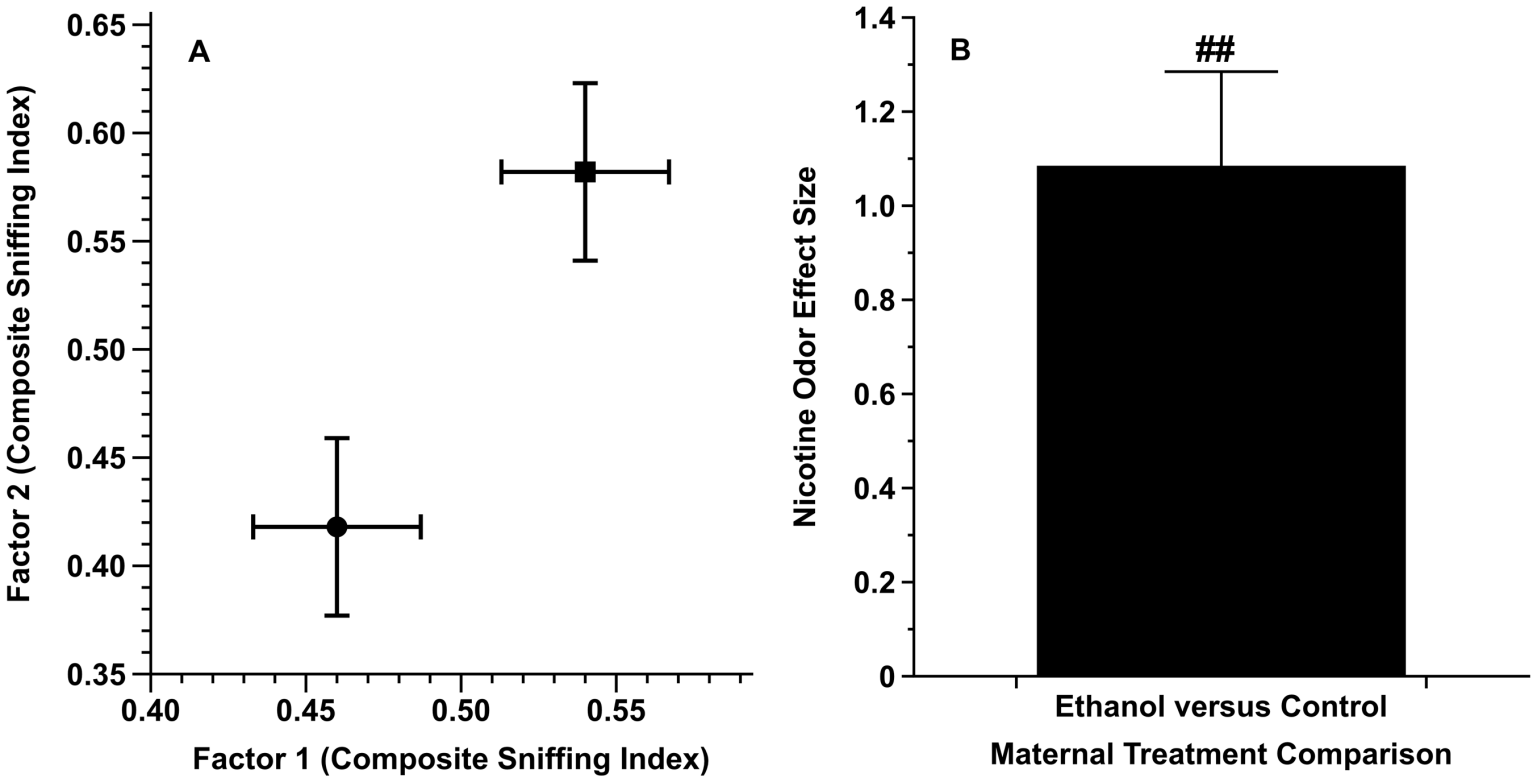
The consequence of prenatal alcohol exposure on the innate odor-mediated behavioral response to nicotine. Panel A illustrates the comparative location of the prenatal alcohol- versus control-exposed groups in a nicotine odor-mediated behavioral response space. The data points are the adjusted least square mean sniffing indexes (± two-dimensional se) as a function of the two prenatal treatments (Solid circles = alcohol exposed; Solid squares = control exposed). Panel B shows the nicotine response-mediated weighted effect size (mean ± se) calculated from the data illustrated in Panel A. ## = *P*<0.03; see text for details.

Figure 1A shows the comparative location of the ET versus FCL animals in a nicotine odor-mediated behavioral response space. In this two-dimensional figure, the degree to which the ET and FCL animals' behavioral responses to the odor of nicotine were equivalent plots as their proximity to each other in the graph (e.g., [33], [59], [60]). As seen qualitatively, on average, there was an unambiguous separation between the two maternal treatment groups, thereby suggesting a degree of difference in maternal alcohol treatment on the inborn behavioral response to nicotine odor.

To formally test our *a priori* hypothesis (namely, whether prenatal alcohol exposure impacts the odor-mediated behavioral response to nicotine) a significance test (two-tailed t: *P*<0.05) was accomplished using the combined weighted city-block distance of the effect sizes for the two indexes (i.e., the two dimensions in Fig. 1A) related to the two randomized ET versus FCL maternal treatments [73], [74]. The weighted city-block distance (Fig. 1B) was the summation of absolute values of the effect sizes for the two indexes. As previously described, the weighting scheme for each principal component factor was the excess of its Eigen value above the Kaiser criterion of 1 used in the PCA (*ibid*). This primary test demonstrated that, on average, there was an overall significant consequence of prenatal alcohol exposure on the innate odor-mediated behavioral response to nicotine (t [18] = 2.38; *P*<0.03).

To provide additional meaning to the above overall main effect of maternal treatment, we performed a secondary assessment to explore whether there were any differential effects of sex or sex by treatment interaction. Multivariate analysis of variance (MANOVA) showed no evidence for either source of variation (F [2,25] = 1.32, *P*>0.2 and (F [2,25] = 1.47, *P*>0.2).

### Orosensory-Mediated Nicotine Acceptance

The goal of the second experiment was to determine whether fetal alcohol exposure altered the oral acceptability of nicotine. In this study, recall that we also included an appetitive tastant, sucrose, as previous work has shown no effect of prenatal alcohol on the orosensory acceptability of sucrose [33].

For the brief access lick testing, of the 11 male and 11 female ET and 11 male and 11 female FCL animals trained on the task, all animals participated on the day of sucrose testing. On average, there was no evidence (t [42] = −0.64, *P*>0.5) of a differential effect of maternal treatment on the number of stimulus presentation blocks completed (ET: 6.3±0.37, FCL: 6.7±0.35 [data are the mean ± se]). In short, the animals' mean lick responses were based on approximately 6.5 trials per stimulus level presented. For the nicotine testing, only 19 ET and 19 FCL animals contributed to the data set. Six animals were eliminated (ET: 2 female, 1 males, FCL: 1 female and 2 male) because of unstable responding across stimulus presentations (i.e., highly variable motivation). On average, there was no evidence (t [36] = −1.08, *P*>0.2) of a differential effect of maternal treatment on the number of stimulus presentation blocks completed (ET: 4.4±0.14, FCL: 4.67±0.18 [data are the mean ± se]). Thus, the animals' mean lick responses were based on approximately 4.5 trials per stimulus level presented.

**Figure 2:**
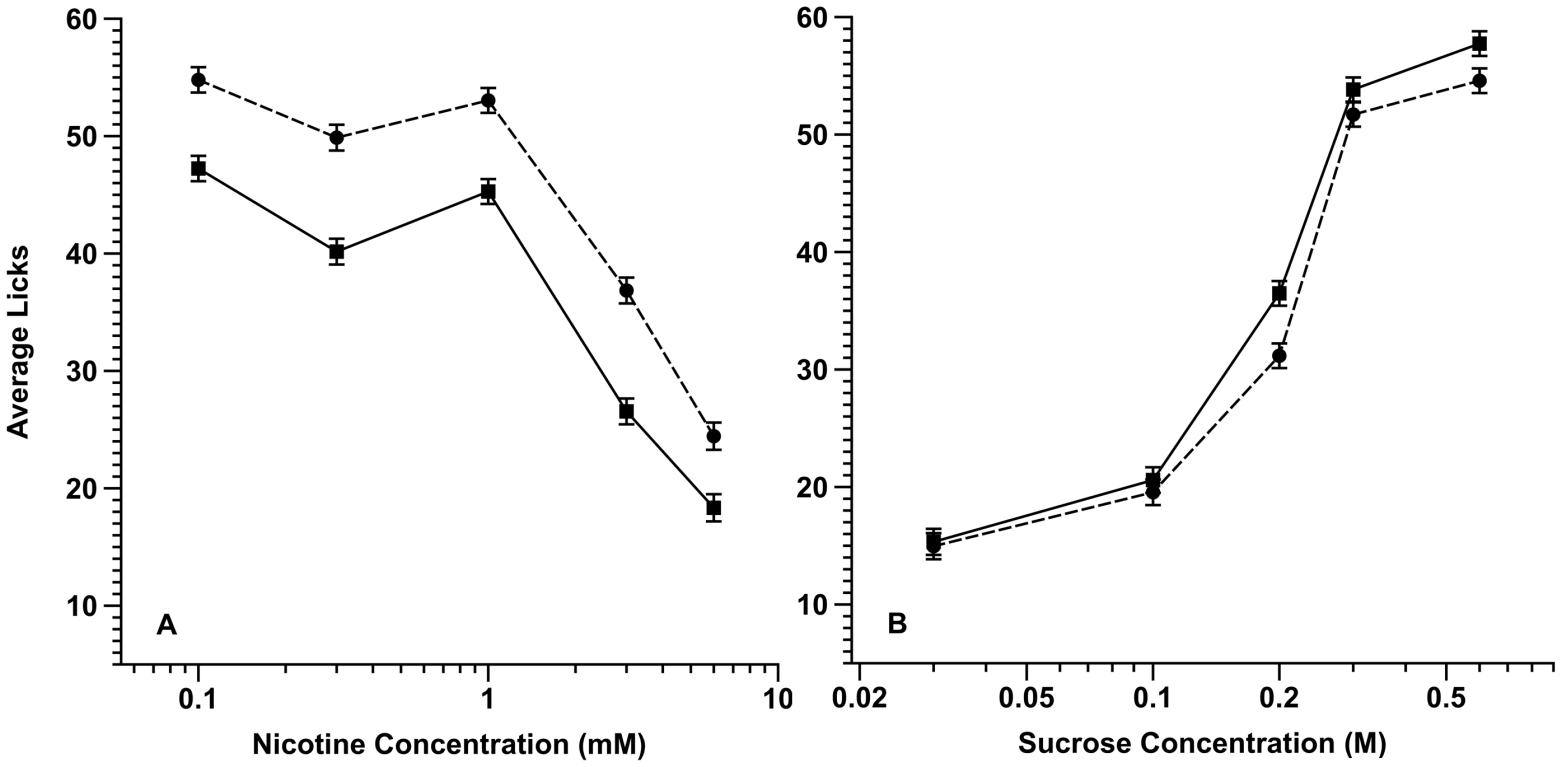
Oral acceptability of a concentration range of nicotine (A) and sucrose (B) solutions to prenatal alcohol- and control-exposed rats. Prenatal alcohol increased the acceptability of nicotine (A) and but not sucrose (B). The data points are expressed as the adjusted least square mean average licks (± se). Note: the scale on the x-axis of A and B differ. Solid circles = alcohol exposed; Solid squares = control exposed.

Figure 2 (Panels A and B) illustrates the average licking rates of adolescent rats as a function of maternal treatment across a range of nicotine and sucrose concentrations, respectively. With respect to nicotine (Fig. 2A), animals in both treatment groups showed a parallel non-linear concentration-dependent decrease in the average number of licks with increasing concentration that was relatively flat between 0.1 to 1 mM nicotine and then decreased sharply thereafter. Nonetheless, there were obvious effects of maternal treatment. By contrast, for the control tastant, sucrose (Fig. 2B), as expected (*ibid*) animals in each prenatal exposure group showed, for the most part, coincident concentration-dependent increases in licking that had a dynamic range between 0.03 and 0.3 M sucrose, plateauing thereafter.

In this study, the lick values to the five nicotine or sucrose concentrations represented a set of correlated variables in a repeated measures design. Because of the non-linear relationship among all pairs of the dependent variables (especially nicotine) the natural log transformed lick data were used to evaluate the main effect of ET vs. FCL maternal treatment (a between factor) and concentration (a within factor) (ANOVA; α<0.05).

For nicotine, there was a significant effect of both prenatal treatment (F [1,33] = 4.27, *P*<0.05) and concentration (F [4,132] = 3.15, *P*<0.02). There was no differential sex effect [F [1,33] = 1.17, *P*>0.2), sex x maternal treatment interaction (F [1,33] = 0.08, *P*>0.7) or concentration x maternal treatment interaction (F [4,132] = 0.38, *P*>0.8). For the tastant, sucrose, there was significant evidence of an overall effect of concentration (F [4,160] = 166, *P* = nil). There was no evidence of an effect of prenatal exposure (F [1,40] = 1.08, *P*>0.3), sex (F [1,40] = 0.20, *P*>0.6), sex x prenatal exposure (F [1,40] = 0.06, *P*>0.8), or prenatal treatment x concentration (F [4,160] = 0.34, *P*>0.8) interactions.

Important to the interpretation of the above, for both the nicotine and sucrose testing we found no evidence of an effect of maternal treatment on the lick responses to water trials. For the aversive tastant nicotine, the ET versus FCL water responses (mean ± se) was 61.3±2.5 and 55.4±2.4, respectively (t [36] = 1.71, *P*>0.09). For the appetitive stimulus sucrose, the ET versus FCL water responses (mean ± se) was 13.6±1.7 and 17.2±2.4, respectively (t [36] = −1.09, *P*>0.2).

To provide additional interpretability to the above results, we performed exploratory analyses on the latency to respond to water within each tastant specific testing session as well as each tastant across the appropriate concentration series. With respect to water trials, we found no evidence of an effect of maternal treatment under the conditions of either nicotine (ET: 4.77±0.77, FCL: 6.4±1.27, t [36] = −1.12, nominal *P*>0.2) or sucrose (ET: 10.49±1.80, FCL: 10.65±1.46, t [36] = −0.06, nominal *P*>0.9) testing.

**Figure 3:**
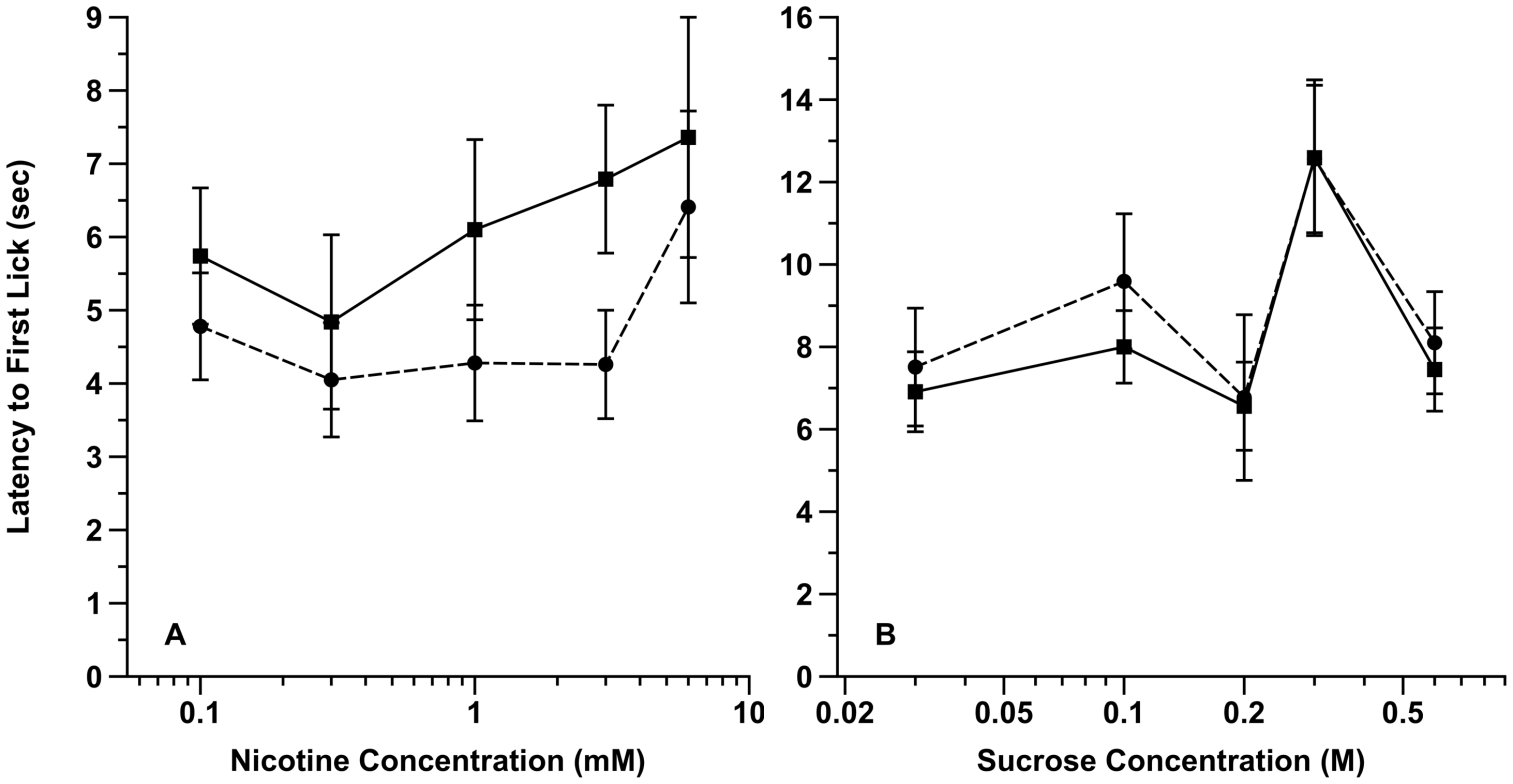
Latency to first lick response for a concentration range of nicotine (A) and sucrose (B) solutions by prenatal alcohol- and control-exposed rats. Compared to control, prenatal alcohol animals responded faster to nicotine (A), but not sucrose (B). The data is expressed as the average latency (± se). Note: the scale on the x-axis of A and B differ. Solid circles = alcohol exposed; Solid squares = control exposed.

Figure 3 (Panels A and B) illustrates the response latency of adolescent rats as a function of maternal treatment for the nicotine and sucrose concentrations, respectively. With respect to nicotine (Fig. 3A), the latency to respond to each stimulus concentration was, on average, faster in the ET animals. Exploratory ANOVA based on observational error found evidence for a significant overall differential effect of maternal treatment (F [1,178] = 3.91, nominal *P*<0.05) and sex (F [1,178] = 4.14, nominal *P*<0.05), with no evidence for an overall effect of concentration (F [4,178] = 1.38, nominal *P*>0.2), sex x maternal treatment (F [1,178] = 0.10, nominal *P*>0.7) and maternal treatment x concentration (F [4,178] = 0.24, nominal *P*>0.9) interactions. For sucrose, although there was evidence for an average effect of concentration on the animals' response latencies (F [4,208] = 5.23, nominal *P*<0.001), there was no evidence of an effect of treatment (F [1,208] = 0.42, nominal *P*>0.5), sex (F [1,208] = 1.24, nominal *P*>0.2), sex x treatment (F [1,208] = 0.04, nominal *P*>0.8) or concentration x treatment (F [4,208] = 0.09, nominal *P*>0.9) interactions

In total, the data revealed that prenatal alcohol exposure reduced the aversive response to nicotine across a range of concentrations while, as expected, not altering the appetitive response to sucrose in adolescent rats (*ibid*).

## Discussion

The negative effects in neural development associated with fetal alcohol exposure are many, encompassing a range of neurobehavioral and developmental consequences ranging from maladaptive behaviors to defects in cognitive function (see rev [76]). Human studies further demonstrate that gestational exposure increases the chance of adolescent alcohol consumption, tobacco product use, and even the use of illicit drugs [6], [7], [77].

The potential importance of the relationship between prenatal alcohol exposure and the co-morbid expression of alcohol and tobacco use, and abuse, cannot be overstated. In terms of the initiation phase of drug use, both alcohol and tobacco products are, at a minimum, the earliest potentially addictive substances generally used by children and early teens [11], [78], [79]. Importantly, human studies demonstrate the younger the involvement with alcohol the more likely the chance of lasting misuse [6], and the initiation and maintenance of smoking behavior in adolescence has been shown to be positively associated with alcohol consumption [17]. The co-morbid use of alcohol and smoking shows a monotonic relationship during adolescence and through young adulthood (*ibid*). Alcohol and tobacco product use are also so called “gateway drugs” that precede the subsequent use and abuse of illicit substances [78].

Despite our understanding of how alcohol and nicotine impact similar neural pathways related to drug-taking behavior and reinforcement (see rev [21]), the mechanism(s) that specifically tie prenatal alcohol exposure to both postnatal alcohol and nicotine dependence [6], [7] still remains largely an open question. On the one hand, as suggested by the broader co-morbidity literature, it may be the case that the observed association is a function of genetic and environmental risk factors: encompassing an array of mechanisms ranging from changes in gene expression affecting the regulation of specific brain neurotransmitters to the impact of fetal alcohol exposure on psychosocial factors. On the other hand, there may be consequences of fetal alcohol exposure that directly impact the initial risk of choice behavior and acceptance for both alcohol and nicotine, thereby priming the adolescent system to be augmented by the aforementioned other factors.

Regarding the above, the flavor attributes of both alcohol [30] and nicotine [31], [32] are key contributors to their acceptance. Alcohol and nicotine have similar flavor profiles encompassing, in particular, the negative attributes of bitter taste, oral irritation and an aversive odor. Previously, we demonstrated that fetal alcohol exposure increased alcohol acceptability in adolescent animals by decreasing its aversive taste (bitter), oral irritation (burning) and smell attributes [33], [34]. Mechanistically, there is evidence that behavioral consequence of fetal alcohol exposure occurred because of the maternal treatment effect at the level of the orosensory periphery through a decreased expression of *T2r* and *Trp* receptor genes central to the transduction of alcohol's bitter and oral irritating qualities, respectively, as well as the sensory transduction of bitter (more specifically, *Trpm5*) [35]. Fetal alcohol exposure also altered the expression of olfactory bulb genes important for synaptic transmission and plasticity [40]. Given that alcohol and nicotine have common chemosensory attributes, and these, in turn, are received and conveyed through the same receptors and pathways, we hypothesized that fetal alcohol exposure would alter the: (1) odorant-induced innate behavioral response to nicotine odor and (2) orosensory-mediated acceptability of nicotine in adolescent rats. The present study found that fetal alcohol exposure from G6-G21 yielded an altered odor-mediated response that we interpret as either a reduced aversive or an enhanced preference response to nicotine (Fig. 1) that was paralleled by increased oral acceptability of nicotine in adolescent animals (Fig. 2).

With regard to our data interpretation several points need consideration. It should be noted that the plethysmography data does not directly assign a valence to the observed alterations in sniffing. It only identifies whether a main effect of treatment has occurred. Nevertheless, in light of the detailed orosensory-mediated behavioral findings the parsimonious explanation is one of an enhanced valence effect to nicotine odor in conjunction with nicotine-specific increased oral acceptance. First, compared to controls, fetal alcohol exposed animals showed both enhanced lick responses to nicotine solutions across a range of concentrations and a faster latency to respond that was specific to this tastant. With respect to this latter observation the shorter latency to respond could be interpreted as an additional index of either an enhanced nicotine oral acceptability, odor preference or both. At present we cannot distinguish between these potential alternatives. Second, fetal alcohol- and control-exposed animals did not differ in terms of their average lick responses or latency to lick for water under the conditions of both nicotine and sucrose testing. This latter finding both highlights the consequence of fetal alcohol exposure on the response to nicotine and argues against a generalized motor effect in terms of the oral acceptability findings. Finally, the overall specificity of the orosensory-mediated results (i.e., no effect for the high caloric appetitive stimulus sucrose) argues against decreased fetal nutrition as the exclusive or major factor that underlies the enhanced nicotine-acceptance we observed.

Our findings are fundamentally important in several ways. The data extend upon prior fetal alcohol work by providing a broader perspective for the proposal that a mother's drug use can be passed to their children via experience-based chemosensory mechanisms (e.g., [33]). In other words, from an epigenetic perspective [35], [41] our findings provide potential insight into how fetal alcohol exposure may also lead to the enhanced risk for initial nicotine use and continued choice behavior in adolescence [6], [7]. They also speak to a broader concern regarding the association between maternal drug use and postnatal vulnerability to co-morbid choice behavior, as many licit and illicit drugs have prominent chemosensory components. Thus, where common sensory mechanisms are at play, there may be broader implications related to the consequence of fetal exposure with one substance of abuse and the initial acceptability of others.

The observation in the present study that prenatal alcohol altered the behavioral response to nicotine odor stands in contrast to the previous observation that fetal alcohol experience-induced olfactory plasticity was specific to the exposure odorant (i.e., in a prior study the non-fetal-exposure stimulus tested was the fruity smelling chemical ethyl acetoacetate) [65]. Consistent with this prior observation there are a number of studies demonstrating that *in utero* odorant experience, as a function of a mother's diet, leads to stimulus specific preferences for the exposure odorant (e.g., [80]–[84]). As such, the discrepancy in outcomes (i.e., no behavioral odor-mediated effect for ethyl acetoacetate in a prior study [65] vs. a significant effect for nicotine in the present experiment) highlights two mechanistic bases for the fetal exposure effects on olfactory responses. These effects are not mutually exclusive, but rather, their contribution varies with test stimulus.

At least for odor, the available data demonstrate that fetal alcohol experience results in odorant specific imprinting that *may* be independent of epigenetic consequences on the olfactory system, per se [40]. Recently, we demonstrated that fetal-alcohol-induced olfactory behavioral plasticity required the associative pairing of alcohol's odor quality and its reinforcing aspects [74]. That is, gestational treatment of pregnant rats with naltrexone (an opiate antagonist that modulates the reinforcing aspects of alcohol [85]) simultaneously with an alcohol-containing diet significantly reduced the enhanced alcohol odor-mediated behavioral effect in their offspring. Of course, in this same study, we cannot rule out the possibility that naltrexone treatment, through its neuroprotective effects [86], also mitigated the epigenetic consequences of fetal alcohol exposure. Nonetheless, there are epigenetic chemosensory consequences of fetal alcohol exposure [35], [40], [41] whose potential impact on observed olfactory function likely varies as a function of odorant. It is well established that nasal chemoreception is conveyed through a number of neural systems that interact to yield the final percept (e.g., [87], [88]). These systems include, not only, the olfactory nerve (Cranial Nerve I) (e.g., [89]), but also the trigeminal nerve (Cranial Nerve V) (e.g., [42]) and isolated solitary chemosensory cells that are trigeminally innervated (e.g., [44]). The extent to which different odorants differentially stimulate these three systems varies. For example, in a study of human subjects with known anosmia Doty [87] found, at the extremes, no observers were able to detect 2-phenyl ethyl alcohol (considered to be a pure olfactory stimulus), whereas all observers were able to detect the strong trigeminal irritant acetone. Thus, the intersection between the effects of fetal alcohol exposure on specific chemosensory related receptor expression and the extent to which specific inhaled odors stimulate olfactory, trigeminal and solitary chemosensory cells of the nasal cavity likely impacts the observed odorant specificity of the behavioral response. Using ethyl acetoacetate versus nicotine as a case in point, at least to humans, ethyl acetoacetate is a fruity pleasant (i.e., non-irritating) odorant [90] that likely primarily stimulates the olfactory system [87]. Thus, the specificity of the previously observed odor-mediated effect in fetal alcohol exposed rats relative to ethyl acetoacetate is not surprising [65]. By contrast, nicotine's irritating and aversive odor [48]–[51] is perceived through olfactory, trigeminal (*Trpv1*-mediated) and solitary chemosensory cell (*T2r- and Trpm5*-mediated) pathways [42]–[46], [48], [50]–[52] with the later two predominating. Given the observation of decreased *T2rs*, *Trpv1* and *Trpm5* expression in the oral cavity of fetal alcohol exposed animals it is reasonable to expect these same genomic effects in the nasal cavity where both *Trpv1* (nasal trigeminal: e.g., [42], [43]) and, *T2r* and *Trpm5*-expressing solitary chemosensory cells [44] respond to irritants such as nicotine. As such, the finding in the present study of an altered response to nicotine odor is also not surprising.

## Conclusions

Given the common aversive component qualities imbued in the flavor profiles (odor, taste and oral irritation) of both alcohol and nicotine, our findings demonstrate that like postnatal alcohol acceptance, fetal exposure to alcohol also influences nicotine acceptability, at a minimum, by decreasing the aversive properties of both its smell and taste. In doing so, our findings suggest a potential chemosensory based mechanism by which fetal alcohol exposure increases the later initial risk for nicotine abuse, thereby contributing to the co-morbid expression with enhanced alcohol consumption. At a more general level, the present findings point to a broader mechanistic concern regarding the consequence of fetal exposure with one substance of abuse and its impact on the potential initial acceptability of others, as many licit and illicit drugs have prominent chemosensory components with likely common underling sensory transduction pathways.
